# BK virus encephalopathy and sclerosing vasculopathy in a patient with hypohidrotic ectodermal dysplasia and immunodeficiency

**DOI:** 10.1186/s40478-016-0342-3

**Published:** 2016-07-13

**Authors:** Armine Darbinyan, Eugene O. Major, Susan Morgello, Steven Holland, Caroline Ryschkewitsch, Maria Chiara Monaco, Thomas P. Naidich, Joshua Bederson, Joanna Malaczynska, Fei Ye, Ronald Gordon, Charlotte Cunningham-Rundles, Mary Fowkes, Nadejda M. Tsankova

**Affiliations:** Department of Pathology, Icahn School of Medicine at Mount Sinai, New York, 10029 NY USA; Laboratory of Molecular Medicine and Neuroscience, National Institute of Neurological Disorders and Stroke, National Institutes of Health, Bethesda, 20892 MD USA; Department of Neurology, Icahn School of Medicine at Mount Sinai, New York, 10029 NY USA; Department of Neuroscience and Friedman Brain Institute, Icahn School of Medicine at Mount Sinai, 1425 Madison Avenue, Icahn 9-20E, New York, 10029 NY USA; Laboratory of Clinical Infectious Diseases, National Institute of Allergy and Infectious Diseases, National Institutes of Health, Bethesda, 20892 MD USA; Department of Radiology, Icahn School of Medicine at Mount Sinai, New York, 10029 NY USA; Department of Neurosurgery, Icahn School of Medicine at Mount Sinai, New York, 10029 NY USA; Department of Medicine - Allergy & Immunology, Icahn School of Medicine at Mount Sinai, New York, 10029 NY USA

**Keywords:** Polyomavirus, BK virus, IKK-gamma, NF-kappa-B essential modulator (NEMO), Ectodermal dysplasia, HED-ID, Encephalopathy, Fibrosing vasculopathy

## Abstract

Human BK polyomavirus (BKV) is reactivated under conditions of immunosuppression leading most commonly to nephropathy or cystitis; its tropism for the brain is rare and poorly understood. We present a unique case of BKV-associated encephalopathy in a man with hypohidrotic ectodermal dysplasia and immunodeficiency (HED-ID) due to IKK-gamma (NEMO) mutation, who developed progressive neurological symptoms. Brain biopsy demonstrated polyomavirus infection of gray and white matter, with predominant involvement of cortex and distinct neuronal tropism, in addition to limited demyelination and oligodendroglial inclusions. Immunohistochemistry demonstrated polyoma T-antigen in neurons and glia, but expression of VP1 capsid protein only in glia. PCR analysis on both brain biopsy tissue and cerebrospinal fluid detected high levels of BKV DNA. Sequencing studies further identified novel BKV variant and disclosed unique rearrangements in the noncoding control region of the viral DNA (BKV_N_ NCCR). Neuropathological analysis also demonstrated an unusual form of obliterative fibrosing vasculopathy in the subcortical white matter with abnormal lysosomal accumulations, possibly related to the patient’s underlying ectodermal dysplasia. Our report provides the first neuropathological description of HED-ID due to NEMO mutation, and expands the diversity of neurological presentations of BKV infection in brain, underscoring the importance of its consideration in immunodeficient patients with unexplained encephalopathy. We also document novel BKV_N_ NCCR rearrangements that may be associated with the unique neuronal tropism in this patient.

**Electronic supplementary material:**

The online version of this article (doi:10.1186/s40478-016-0342-3) contains supplementary material, which is available to authorized users.

## Background

Here we report the first histological description of BK virus encephalopathy with cortical predominance in a male patient with hypohidrotic ectodermal dysplasia and immunodeficiency (HED-ID), a rare X-linked disorder due to mutation in the NFkB signaling pathway. Ectodermal dysplasias are a group of inherited disorders characterized by absence or dysplasia of ectodermal appendages. In the hypohidrotic form, there is abnormal development of eccrine sweat glands, teeth, and hair [23]. Mutations within a regulatory subunit of the NFkB pathway, IKK-gamma/NF-kappa-B essential modulator (NEMO), are known to cause two distinct X-linked forms of ectodermal dysplasia: X-linked dominant familial incontinentia pigmenti (IP), which is typically fatal prenatally in males; and X-linked recessive disorder with rare combination of HED and immunodeficiency (HED-ID), in which incomplete loss of NEMO function leads to impaired immune response with recurrent bacterial and viral infections in addition to HED [2, 17, 21, 30, 46, 50, 59]. Herein, we demonstrate BKV encephalopathy by histology, immunohistochemistry, sequencing, and further characterize a novel BKV variant (BKV_N_), which harbors unique rearrangements in the noncoding control region (NCCR), potentially contributing to its neuronal tropism. Finally, we describe several other previously unreported neuropathological features in this patient in the context of his underlying HED-ID disorder.

Polyomaviruses are non-enveloped, small (~40 nm) viruses with a circular double-stranded DNA genome. BK polyomavirus (BKV) displays approximately 75 % DNA homology with JC polyomavirus (JCV) and 70 % homology with the simian polyomavirus SV40. Infection with BKV occurs during childhood and is usually asymptomatic. Thereafter, the virus enters a state of latency, predominantly in the renal tubular epithelial cells, during which BKV DNA can be detected, while viral proteins such as T-antigen, agnoprotein and capsid proteins, cannot. By adulthood, 80–90 % of the general population is persistently colonized with BKV [31, 32]. Reactivation of the latent virus occurs predominantly in patients with impaired immune function, including HIV-1/AIDS, lymphoproliferative disorders and other malignancies, and treatment with immunosuppressive drugs [10, 48]. The most common pathologic conditions associated with reactivation of BKV are polyomavirus-associated nephropathy or PVAN in kidney transplant recipients [13, 14, 52] and hemorrhagic cystitis in bone marrow transplant recipients [3, 31]. Less often, BKV is reported to cause fatal pneumonia, retinitis, native kidney nephritis, and only rarely meningoencephalitis in the severely immunocompromised [12, 16, 54]. JC polyomavirus, rather than BK, is by far the most commonly associated virus with a CNS tropism in the context of immunosuppression, causing a progressive multifocal demyelinating leukoencephalopathy (PML) [29].

## Case presentation

A 29-year-old man had been diagnosed with immunodeficiency at the age of one year after developing recurrent bacterial and viral infections in the setting of dysgammaglobulinemia with low levels of IgG. He also displayed signs of ectodermal dysplasia, including conical-shaped incisors, hypodontia and inadequate sweating. After multiple recurrent infections, at the age of 12, the patient was diagnosed with HED-ID caused by NEMO deficiency. Genomic analysis revealed a missense mutation within the putative zinc-finger domain in the most 3′ exon of *IKK-gamma* - exon 10, causing a cysteine to arginine substitution [2, 59]*.* Notably, the patient’s older brother had very similar clinical presentation and passed away at the age of 17 with recurrent bronchiectasis after bilateral lung transplantation. Following diagnosis of HED-ID in the reported patient, lifelong IVIG treatment was initiated and maintained, leading to stabilization of IgG levels and a normal quality of life.

**Fig. 1:**
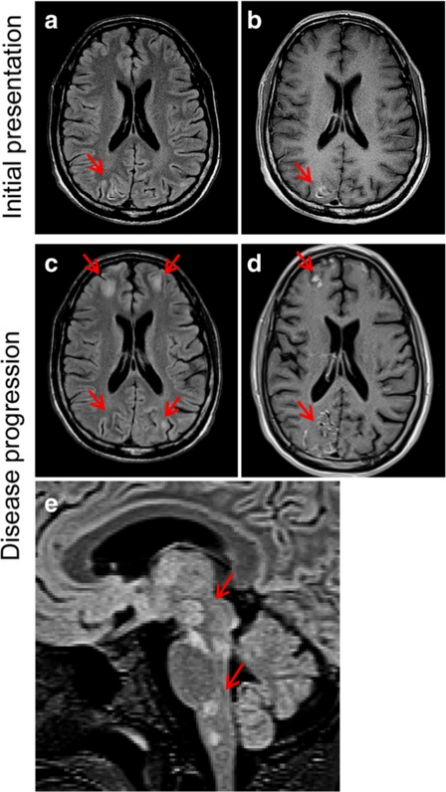
Serial axial T2 FLAIR MRI (**a**, **c**) and corresponding axial contrast-enhanced T1 MRI (**b**, **d**) imaging of patient during initial presentation and after three months of disease progression when biopsy was performed. **a** Initial MRI (Day 1 of hospitalization) shows cortical gray matter with increased T2 FLAIR signal that is most prominent in the right occipital lobe (arrow) and just appreciable in the left occipital lobe. The related sulci are prominent, not compressed. The juxta-cortical white matter appears normal. **b** There is prominent contrast enhancement of portions of the affected cortices with no leptomeningeal enhancement. **c**-**d** Surveillance axial T2 FLAIR MRI (**c**, Day 90 of hospitalization,) and contrast-enhanced T1 MRI (**d**, Day 82 of hospitalization,) show disease progression with prominent involvement of both occipital and both frontal lobes. **e** T2 MRI through the deep gray matter and brain stem (sagittal) on Day 81 of hospitalization demonstrates patchy regions with signal abnormality in the basal ganglia, thalami and brain stem, including the medial left thalamus, right lateral geniculate nucleus, inferior colliculus, interpeduncular nuclei, dorsal pontine nuclei, and lateral medullary nuclei

At the age of 29, the patient experienced new-onset neurological symptoms including left homonymous hemianopsia and intermittent, pressure-like headache, without significant motor, sensory, or cognitive impairment. Initial MRI of the brain (Fig. 1a, b) showed multiple scattered foci of increased T2 FLAIR signal, restricted diffusion, and contrast enhancement, predominantly within the right occipital lobe (likely contributing to the patient’s visual changes). Additional small foci of increased T2 FLAIR signal were seen within the left frontal lobe, left thalamus, left pons and medulla. The varied, bilateral sites of radiographic abnormality suggested a possible embolic process, but infectious and non-infectious inflammatory processes were also considered in the differential. Extensive imaging workup failed to reveal an embolic source, and cultures of blood, urine, and cerebrospinal fluid (CSF) did not demonstrate bacterial, viral or fungal organisms. The patient’s neurological status continued to deteriorate over the next 3 months with progressive disorientation and cognitive decline. Follow-up MRIs now showed further disease progression with more extensive involvement of both occipital lobes, prominent involvement of the cortex and subcortical white matter of both frontal lobes (Fig. 1c, d), and expanded involvement of the left thalamus and brainstem (Fig. 1e). CSF studies remained negative for bacteria, fungal elements, and a variety of viral pathogens (adenovirus, enterovirus, HSV1, HSV2, CMV, VZV, EBV, HHV6, and West Nile, East Equine encephalitis, and St. Louis encephalitis arboviruses). CSF PCR for JCV was negative on two separate occasions. Approximately three months after initial presentation, a targeted right occipital brain biopsy was performed for definitive diagnosis.

**Fig. 2:**
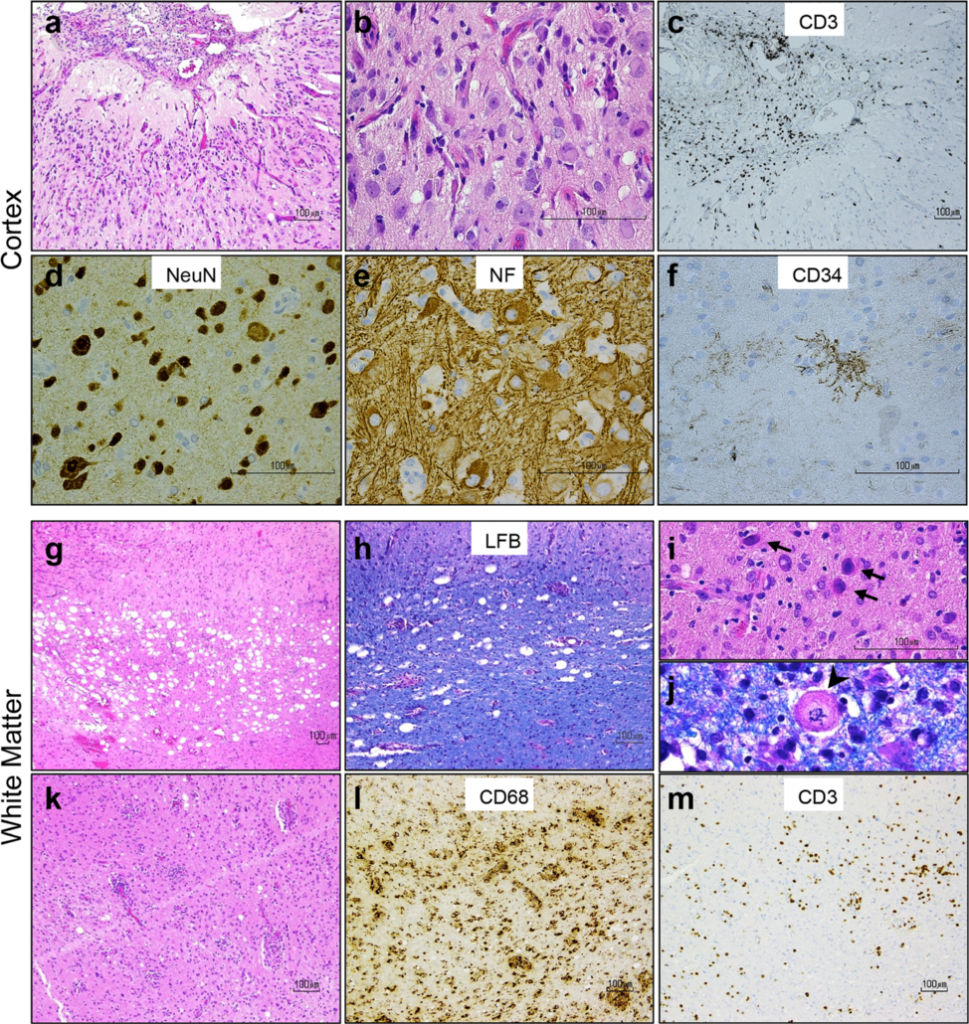
Histological analysis of right occipital brain biopsy reveals unusual form of inflammatory encephalopathy. **a**-**b** Hematoxylin and eosin (H&E) stains show chronic meningitis and neocortex with architectural neuronal disorganization, associated with reactive vasculature, gliosis, microglial activation, and mild chronic inflammation. **c** CD3+ T-lymphocytes predominate in the meninges. **d** Many cortical neurons display dysmorphic features, such as increased size, displaced Nissl substance, clustering, and **e** abnormal phosphorylated neurofilament accumulation in their cell body. **f** Tuft-like ramified CD34-positive processes are also seen in the neocortex. **g**-**j** The subcortical white matter contains multifocal vacuolization associated with minimal myelin loss on luxol fast blue (LFB) stain, and scattered bizarre oligodendroglial-like cells with enlarged nuclei containing glassy, homogeneous nuclear inclusions (**i**, arrows) and Creutzfeldt-like astrocytes (**j**, arrowhead). **k-m** Prominent chronic perivascular and intraparenchymal inflammatory infiltrate in the white matter is composed of CD68+ macrophage/microglia and CD3+ T-lymphocytes

Histological examination of the biopsy specimen revealed a chronic inflammatory process involving the leptomeninges, underlying cortex and white matter (Fig. 2). The neocortex appeared distinctly abnormal, with widespread architectural neuronal disorganization, vacuolization most prominent in the external pyramidal layer III, reactive vasculature, astrogliosis and microglial activation (Fig. 2a-f). Many cortical neurons showed bizarre dysmorphic features, including increased size, displaced Nissl substance, vacuolization, and clustering (Fig. 2b, d), abnormal neurofilament accumulation in the cell body (Fig. 2e), and occasionally tuft-like ramified CD34-positive processes (Fig. 2f). In the subcortical white matter there was robust multifocal vacuolization (Fig. 2g-j) with minimal associated myelin loss (Fig. 2h, j), and a prominent chronic perivascular and intraparenchymal inflammatory infiltrate composed of CD68+ macrophage/microglia and CD3+ T-lymphocytes (Fig. 2k-m). Scattered bizarre oligodendroglial-like cells with enlarged nuclei were noted, frequently containing glassy, homogeneous nuclear inclusions (Fig. 2i, arrows); Creutzfeldt-like astrocytes were also seen (Fig. 2j, arrowhead).

**Fig. 3:**
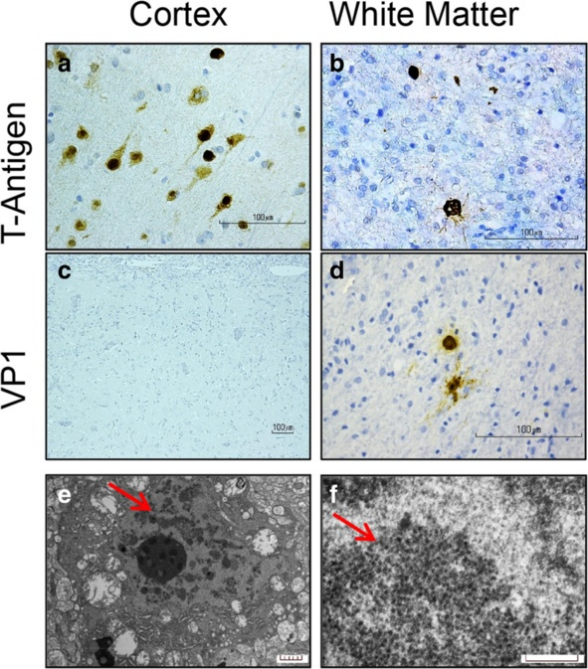
Polyomavirus infection in cortex and white matter by immunohistochemistry and electron microscopy. **a**-**b** Immunostaining with the cross-reacting polyomavirus antibody anti-SV40 T-Antigen shows strong nuclear positivity in cortical neurons, predominantly in layers II-V (**a**) and in scattered enlarged oligodendroglial and astrocytic nuclei (**b**) in the white matter. **c**-**d** Immunoreactivity with the cross-reacting antibody anti-VP1 capsid protein is absent in cortical neurons (**c**) but is present in scattered cells with glial appearance in the white matter (**d**). **e** Electron micrographs reveal a neural cell with aggregates of spherical particles admixed with chromatin (arrow), scale bar = 5 μm. **f** At higher power, 30–40 nm in diameter non-enveloped icosahedral viral particles are observed, focally forming sheets and paracrystalline arrays (arrow); scale bar = 0.26 μm

Immunohistochemical studies with anti-SV40 T-Antigen, which cross-reacts with JC and BK virus T-antigens, showed strong nuclear positivity in cortical neurons, predominantly in layers II-V (Fig. 3a) as well as in scattered enlarged oligodendroglial and astrocytic nuclei with bizarre multilobated appearance (Fig. 3b). The predominant morphology of infected cells in the cortex resembled pyramidal neurons, although infection of other neuronal cell populations could not be excluded. SV40 T-antigen was not identified in the leptomeninges, which otherwise appeared markedly fibrotic and expanded by chronic inflammatory infiltrate (Fig. 2a, c).

We also analyzed expression of the polyomavirus VP1 capsid protein using an antibody that cross-reacts with VP1 of JCV, BKV and SV40. Intriguingly, the number of cells positive for VP1 was significantly lower compared to T-antigen positive cells in the same region (Fig. 3c-d). Noticeably, VP1-immunoreactivity was associated with cells of oligodendroglial or astrocytic phenotype (Fig. 3d) but was largely absent in infected neurons (Fig. 3c). Electron microscopy confirmed the presence of 30–40 nm non-enveloped icosahedral viral particles within the nuclear chromatin, consistent with polyomavirus (Fig. 3e-f).

Detection of JC virus DNA by PCR, the most common polyomavirus to infect the brain, was unsuccessful both in CSF prior to the brain biopsy as well as in the pathological cortical tissue. We therefore considered the possibility of infection by other polyomavirus species, and performed targeted qPCR for BKV at the CLIA/NINDS laboratory [45], which detected a high titer of BK virus within the CSF and biopsy tissue.

**Fig. 4:**
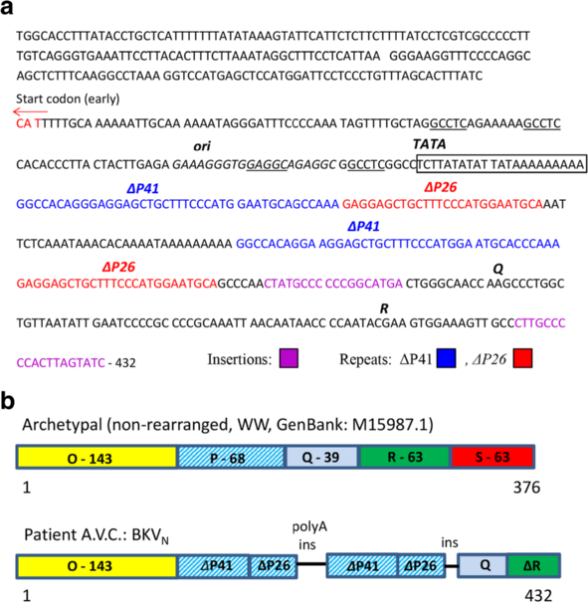
Sequencing confirms BK virus infection in brain tissue and identifies unique BKV_N_ NCCR region. **a** Sequence analysis of BKV_N_ NCCR region. Start codon for the late coding region is shown by red arrow. The origin of viral replication (*ori*) and TATA box are depicted. T-Ag binding sites are underlined. The BKV_N_ P blocks appear in two variants consisting of 41 (ΔP41) and 25 (ΔP26) nucleotides are shown in blue or red, respectively. Each of these blocks is similar to the initial portion of P block of archetype. Insertions/variations are also highlighted (pink). **b** Schematic comparison of NCCR sequence between BKV archetypal and BKV_N_. The NCCR region of the archetypal BKV contains five sequence blocks denoted O-143 (with *ori)*, P-68, Q-39, R-63 and S-63 where the numbers indicate base pairs. The BKV_N_ NCCR contains block O-143, two tandem repeats composed of portions of block P (ΔP41 and ΔP26) and separated by polyA stretch. Blocks Q and R follow the second ΔP26

The double-stranded circular DNA of the BKV genome contains approximately 5100 base pairs arranged around the cellular histones and is comprised of three parts: the early and late coding regions for regulatory and structural proteins that are transcribed in opposite directions, and a bidirectional NCCR located between the early and late coding regions. The early coding region encodes the regulatory proteins large and small tumor antigens, the truncated tumor antigen and a pre-miRNA for two miRNAs [1, 47]. The late coding region encodes the regulatory agnoprotein and the three structural capsid proteins VP1, VP2 and VP3. The NCCR controls the initiation of viral DNA synthesis and regulates the transcription of early and late promoters [18, 57]. To further analyze the VP1 and NCCR regions of our specific BKV variant for sequence motifs in the context of its unique neuronal tropism, we performed a two-step nested PCR amplification followed by Sanger sequencing of BKV VP1 and NCCR (Fig. 4, Additional file 1: Figure S1). The major capsid protein of BK virus, VP1, is involved in the interaction with host cellular receptors [42], but whether specific VP1 polymorphisms are linked to neurological sequelae of BKV is not clear. The NCCR region of BKV contains binding motifs for numerous cellular transcription factors (TFs) regulating viral transcription and replication [11, 36, 57].

Sequence analysis of the patient’s VP1-amplified fragment (Additional file 1: Figure S1) showed high homology (within a few single nucleotide variations) with BKV isolate RU15 (GenBank: FR720320.1; 2153 - 2583 nucleotides) and BKV Dunlop strain (GenBank: V01108.1; 2272 - 2697 nucleotides). The NCCR amplified fragment containing the initial portion of early genes (203 bp with start codon for large T-antigen) also showed high similarity to the Dunlop strain.

We also compared the sequence of our entire NCCR amplicon (432 bp) with the archetypal variant (non-rearranged, WW, GI:4838415). In the most frequently seen archetype BKV strain, NCCR has been arbitrarily divided into five sequence blocks denoted O-143, P-68, Q-39, R-63 and S-63 where the numbers indicate base pairs [40, 41]. Block O contains the origin of replication (*ori)* and binding sites for large T-antigen and TFs, including NFkB, CEBPβ and SP1 [57]. The early and late gene promoters and enhancers are located in P, Q, R, and S blocks. The BKV NCCR in our patient harbored significant deviations from reported NCCR sequences, including four repeats of portions of block P (two ΔP41 and two ΔP26) in addition to a small insertion, deletions and single nucleotide variations. Block O, in contrast, was conserved (Fig. 4). The P blocks in our patient appeared in two variants consisting of 41 (ΔP41) and 25 (ΔP26) nucleotides. Each of these blocks encompassed the initial portions of P block of archetype. The P blocks were arranged in the alternating manner: ΔP41 + ΔP26 followed by small adenine-rich fragment and the second ΔP41 + ΔP26. After the second ΔP26, blocks Q and R were present, although with nucleotide variations and deletions. Block S was not identified, although we cannot exclude the possibility that this region failed to amplify (Fig. 4b). We also compared our sequence with previously reported rearranged BKV isolates and observed the highest homology with NCCR of BKV strains WWM13 (GenBank: JQ513604.1) and WWM9 (GenBank: JQ513600.1). Intriguingly, these strains were isolated from the CSF of patients with neurological symptoms [4], implicating a possible association between these BKV rearrangements and predilection for CNS infection.

**Fig. 5:**
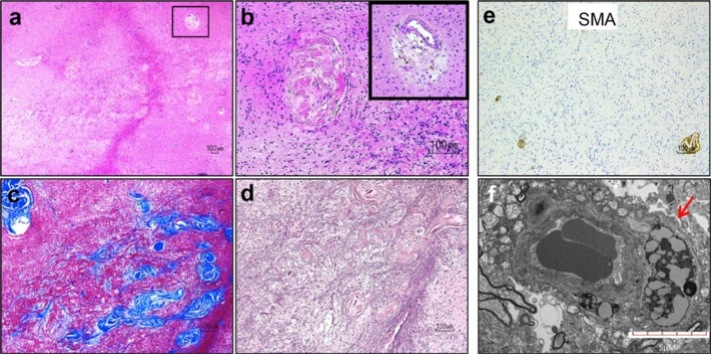
Unusual form of sclerosing vasculopathy in HED-ID. **a**-**b** Hematoxylin and eosin stain shows vessels in the white matter with variably obliterated lumina, hyalinization, and perivascular hemosiderin deposits (inset in **b**). **c**-**d** Concentric lamellated pattern of collagen accumulation is seen on trichrome (**c**), extending into the adjacent brain parenchyma and forming a reticulin-rich scar (**d**). **e** Sclerotic vessels lack smooth muscle fibers in their walls. **f** EM shows capillary vessel with aberrant accumulation of lysosomes (arrow); scale bar = 5 μm

Detailed neuropathological analysis also demonstrated an unusual form of sclerosing vasculopathy in the white matter (Fig. 5), adjacent to but not involved by areas of inflammation and viral infection. We noted several thickened vessels whose walls were entirely replaced by concentric layers of collagen with uniquely associated perivascular lamellar fibrosis (Fig. 5d-e). These vessels lacked smooth muscle fibers in their walls (Fig. 5c), and their lumina were often completely obliterated with attempt at recanalization (Fig. 5b). The vascular fibrosis showed unusual extension into the adjacent brain parenchyma, revealing a dense, reticulin-rich scar (Fig. 5e) almost completely devoid of axonal processes. This angiocentric sclerosing process appeared on a continuum, with some vessels outside of the end-stage scar lesion showing milder collagen deposition with preserved lumina and smooth muscle walls, but with perivascular hemosiderin deposition, indicative of compromised vascular wall integrity (Fig. 5b, inset). Small caliber vessels with aberrant accumulation of lysosomal material were identified on electron microscopy (Fig. 5f, arrow).

## Discussion

We report a very unusual presentation of BK virus encephalopathy in a patient with rare immunodeficiency and ectodermal dysplasia due to NFkB dysfunction and provide the first documented neuropathological description of this rare disorder, including the presence of unique form of sclerosing vasculopathy.

To our knowledge, this is the first histological demonstration that BK virus can infect cortical neurons in man, causing overwhelming encephalopathy with minimal demyelination. CNS infection by BK virus is unusual and clinically under-recognized, although a review of the literature reveals descriptions of BKV-associated encephalitis [7–9, 24, 35, 38, 55], meningoencephalitis [12, 51, 53, 54], PML [15, 19], and retinitis [12, 26]. A single report describes the neuropathological alterations caused by BKV in the brain as marked perivascular and leptomeningeal lymphocytic infiltration and patches of demyelination and gliosis [38]. These elements were also present in our patient. Previous reports based on neuroimaging studies suggest that BKV infection has a predilection for the periventricular and pial surfaces of the brain parenchyma, with the cortex being spared [37]. Our case clearly demonstrates that BKV can also infect cortical neurons leading to progressive encephalopathic symptomatology, with only minimal demyelinating component at the time of biopsy. Indeed, numerous pyramidal cortical neurons in the occipital cortex of our patient showed strong positivity for polyomavirus T-antigen but lacked staining for VP1 capsid protein. Among polyomaviruses, the most common one to infect the brain is JCV, which typically causes a demyelinating disease with a productive and lytic infection of oligodendrocytes and less commonly astrocytes [10, 20, 44]. Although predominant neuronal infection is rare, it has been reported in cerebellar granule cell neurons as JC virus granule cell neuronopathy (JCV-GCN), which is associated with JCV variants harboring a small deletion in the VP1 capsid protein [22, 33], and in cortical pyramidal neurons as JCV encephalopathy (JCV-E), which is caused by JCV variants containing Agno deletions [58]. Interestingly, in JCV-E more neurons are positive for T-antigen, than for VP1 capsid protein, and it has been suggested that this phenomenon is due to latent, recent, or abortive infection [58]. Similar findings were reported in the occipital and temporal cortices of a 21-year-old patient with common variable immunodeficiency, where the presence of JCV T-antigen and P53, but not VP1 capsid protein, was detected in dysplastic ganglion-like cells [49]. Overall, our findings corroborate that polyomaviruses can infect neurons, and underscore the importance of considering BKV infection in the differential diagnosis of immunosuppressed patients with clinical symptoms of encephalopathy with undetectable JCV in their CSF.

Furthermore, we have identified a new BKV isolate with significant rearrangements in its NCCR, which likely contributed to the reactivation of the virus, in agreement with numerous studies showing that the most rearranged strains with deletions, insertions and duplications in NCCR are associated with increased viral replication [25, 43]. We speculate that the activation of BKV in an unusual host compartment – here the neuronal cell - is dependent on these unique NCCR rearrangements. This novel strain has a quadruple enhancer element containing imperfect duplications of an archetypal P element. One possible mechanism for viral activation and/or neuronal tropism may be through selective amplification of TF motifs in this amplified P NCCR region, such as nuclear factor 1 (NF1) or SP1, leading to transcriptional activation of viral genes necessary for replication and/or neuronal infectivity. Further studies are needed to characterize the exact role NCCR rearrangements may play in BKV activation and neuronal tropism, and the regulatory networks involved, which are beyond the scope of this report.

Our detailed neuropathological analysis uncovered two additional unusual features in the brain of this patient with HED-ID, which may be related to his underlying ectodermal dysplasia. The first one was the widespread presence of dysplastic neurons throughout the occipital cortex biopsy specimen. Many of these neurons showed T-antigen immunoreactivity, suggesting that BKV infection may account for their dysmorphic appearance. Intriguingly, many neurons immunoreactive for large T antigen were also positive for p53, but not for capsid protein VP1. Previous studies have suggested a possible role of T-antigen in the dysplastic, ganglion cell-like change of the JCV infected cortical neurons [49]. However, we found that more neurons appeared dysplastic than showed immunohistochemical evidence of infection, raising the additional possibility of an underlying cortical dysplasia superimposed on polyomavirus infection in this patient. In the absence of previous reports on the neuropathological effect of NEMO or BKV on neurons and without further mechanistic studies, it is not feasible to relate unequivocally the presence of abnormal/dysplastic neurons to either BKV–associated cytopathic effect or to HED-ID. The effect of NEMO deficiency on cortical neuronal development has not been previously scrutinized and it deserves further consideration.

We also uncovered a unique sclerosing vasculopathy. The fibrosing process had notable predilection for medium sized vessels in the white matter, and manifested with obliteration of the vessel lumen and deposition of perivascular collagen in a lamellated pattern, which extended into the surrounding brain parenchyma. As well, abnormal accumulation of lysosomal material was noted within the walls of some small-caliber vessels, which may be secondary to hypoxic stress or alternatively relate to a degenerative process. To our knowledge, the appearance of this angiocentric fibrotic scar has not been previously described in other CNS vasculopathic processes. We believe it represents unique dysplastic pathology related to the patient’s underlying ectodermal dysplasia and herein provide its first neuropathological depiction in a male patient with NEMO-deficiency.

There is very limited number of reports on the pathology of HED-ID. The first comprehensive study to describe pathological findings in 13 patients with HED-ID (biopsies and autopsies) did not disclose any neuropathological abnormalities or features specific to NEMO deficiency [28]. CNS findings in this study included agenesis of the corpus callosum in one child, moderate-to-severe cerebral atherosclerosis and hipopigmented substantia nigra in one adult, a remote cerebral cortical infarct, cortical ribbon defect and invasive cerebral aspergillosis in another adult, and subacute and chronic inflammation in the white matter of one living adult patient, without further histological analyses. Skin biopsies were also analyzed in this study, describing mainly inflammatory pathological findings (granulomatous dermatitis with positive AFB staining, lobular panniculitis, seborrheic keratosis, folliculitis) as well as abnormal vascular calcification in two patients; there was no mention of fibrosing vasculopathy and the presence of abnormal lysosomal accumulations were not assessed. While our report is the first to describe fibrosing vasculopathy in the brain of a patient with HED-ID, abnormalities in CNS vessels have been previously observed in patients with the closely related NEMO-deficiency disorder in females, IP [6]. Brain MR imaging studies in patients with IP have demonstrated findings suggestive of microangiopathy [27, 34, 39]. Interestingly, vascular sclerosis and occlusion with associated fibrotic perivascular changes were reported in the retina of patients with IP [56]. The molecular mechanisms underlying these vascular changes are not known, but dysregulation of the NFkB signaling pathway is likely to play a role.

## Conclusion

BK polyomavirus encephalopathy is not typically considered in the differential diagnosis of patients with neurological symptoms, and may be under-recognized in immunosuppressed patients. We suggest that screening for BKV in the CSF of patients with encephalopathy and undetectable JCV is of clinical importance. Our study provides detailed neuropathological description of the manifestation of BKV CNS infection with distinct neuronal tropism in a patient with NFkB-mediated immunosuppression, expanding its clinical recognition. The identified unique BKV sequence may be important for elucidating the mechanisms of BK viral reactivation and neurovirulence in future studies.

## Materials and methods

### Antibodies

Immunohistochemistry (IHC) was performed with the following antibodies: mouse monoclonal anti-NeuN (A60; MAB377; Chemicon), mouse monoclonal anti-Neurofilament (2 F11, 760-2661, Ventana Medical Systems, Inc.), rabbit polyclonal anti- Glial Fibrillary Acidic Protein (GFAP) (EP672Y; 760-4345, Ventana Medical Systems, Inc.), mouse monoclonal anti-p53 (DO-7, 790-2912, Ventana Medical Systems, Inc.), rabbit monoclonal anti-CD3 (2GV6, 790-4341, Ventana Medical Systems, Inc.), mouse monoclonal anti-CD34 (QBEnd/10, 790-2927, Ventana Medical Systems, Inc.), mouse monoclonal anti-CD68 (KP-1, 790-2931, Ventana Medical Systems, Inc.), SV40 T Ag (v-300; sc-20800, Santa Cruz Biotechnology, Santa Cruz, CA) rabbit polyclonal antibody (raised against amino acids 4-30 mapping near the N-terminus of SV40 T Ag). Anti-VP1 antibody (mouse serum ab 597) was kindly provided by Dr. Kamel Khalili (Department of Neuroscience, Temple University School of Medicine, Philadelphia, US).

### Immunohistochemical staining

Paraffin embedded sections (4 μM) fixed in 10 % formalin were deparaffinized in xylene, with subsequent rehydration in a decreasing gradient of ethanol. Antigen retrieval was performed using cell-conditioning solution 1 (Ventana Medical Systems, AZ) for 60 min at 95 °C. After blocking, sections were incubated with primary antibodies according manufacturer protocols. Sections were washed and incubated with mouse or rabbit secondary antibodies depending on the type of the primary antibody on automated strainers at room temperature (Ventana XT). Immunoreactivity was detected by means of the IVIEW or Ultraview Universal DAB Detection Kits (760-500, Ventana Medical Systems, AZ).

### Electron microscopy

Small pieces of brain tissue were received fixed in 3 % glutaraldehyde in a 0.2 M sodium cacodylate buffer at pH 7.4. The tissue was treated with osmium tetroxide for one hour rinsed in 0.2 M sodium cacodylate buffer and then subjected to dehydration in increasing steps of ethanol through propylene oxide and embedded in embed 812. One-micrometer plastic sections were cut, stained with methylene blue and Azure II, and observed by light microscopy. Representative areas were chosen for ultrathin sectioning. The thin sections were stained with uranyl acetate and lead citrate and photographed with a Hitachi H7650 transmission electron microscope equipped with an SIA digital imaging system.

## DNA isolation and sequencing

### DNA extraction

DNA extraction was performed from 40 μl of fresh frozen brain tissue using Qiagen QIAamp DNA kit (Cat. #51304) and Qiagen ATL Buffer (Cat. #19076) according to the manufacturer’s instructions. RNA Carrier (Qiagen Cat. 1068337) was added to the sample to optimize DNA yield. The DNA was eluted in a volume of 45 μl AE Buffer and its concentration was measured using NanoDrop 2000.

**Nested PCR amplification** of VP1 and NCCR sequences of BKV was performed as previously described [4, 5], with slight modifications. Positions of nucleotides for all primers are shown based on nucleotide sequence in BKV Dunlop strain, V01108.

### Amplification of the BKV VP1 region (nt 1456-2744)

In the first reaction, the following primers were used: forward VP1-F1 5′-AAACTATTGCCCCAGGAGGT-3′ (nucleotides (nt) 1456–1475) and reverse VP1-R4 5′-CTAAAACACCACCCCCAAAA-3′ (nt 2725–2744). A 1289 bp PCR products were purified by gel extraction using Qiagen QIAquick gel extraction kit (Qiagen, Cat. 28704) and used for nested PCR with the following primers: forward VP1-F4 5′-CTAATCAAAGAACTGCTCCTCAATG-3′ (nt 1477–1501) and reverse VP1-R8 5′-ACCACCCCCAAAATAACACA-3′ (nt 2718–2737), producing an amplicon of 1261 bp. Both reactions were performed in a volume of 50 μl with 500 nM of each primer, 5 mM MgCl_2_, 500 μM dNTP’s, and 1.25 U of AmpliTaq Gold (ThermoFisher, Cat. N8080248) and nuclease-free water. The first PCR was performed on 2 μl eluted DNA. A 4 μl of the first PCR product was used in the nested PCR. PCR conditions: denaturation - 15 min at 94 °C, 40 cycles of 30 s at 94 °C, 30 s at 55.7 °C (65 °C for the nested PCR), and 60 s at 72 °C with a final extension of 7 min at 72 °C.

### Amplification of the BKV NCCR region (nt 4881-680)

In the first reaction, the following primers were used: forward ORIBK1 5′-ATCTGGGCAAAGAGGAAAATCA-3′ (nt 4881–4902) and reverse ORIBK2 5′-AGCAGCCTCAGATACACTGG-3′ (nt 661–680). Nested PCR was performed with Forward ORIBK3 5′-CAGGTTCCAAAATCAGGCTG-3′ (nt 4924–4943) and reverse ORIBK 5′-CTAGGAGTCTTTTACA-GAGTCT-3′ (nt 567–588) primers. For both amplification reactions, AmpliTaq DNA polymerase with GeneAmp kit (ThermoFisher Cat. N8080248) including 1.25U of the polymerase were employed. A final volume of 50 μl was completed with 500 nM of each primer, 500 μM of dNTPs, 5 mM of MgCl_2_, nuclease-free water and 2 μl of the DNA eluted (or 4 μl of the first PCR product for the nested PCR). PCR conditions: denaturation 15 min at 95 °C, 40 cycles of 30 s at 94 °C, 30 s at 56 °C (58 °C for the nested PCR), and 1 min at 72 ° with a final extension - 7 min at 72 °C. The amplicons were separated by electrophoresis on a 1 % agarose gel in the presence of ethidium bromide and were visualized under UV light. To avoid contamination, ultrapure reagents were added under a laminar flow hood and in a separate room.

## Consent

Informed consent was obtained from all individual participants included in the study.

## Additional file

Additional file 1: Targeted sequencing strategy for BKV VP1 and NCCR regions. **a**. Products of nested PCR encompassing 1265 bp of BKV VP1 and 800 bp of NCCR with initial portion of early coding region are purified from an 1 % agarose gel and submitted to Sanger sequencing. **b**. Sanger sequencing results of VP1 and NCCR purified gel products. (PDF 427 kb)
