# Supplementary material for: BK virus encephalopathy and sclerosing vasculopathy in a patient with hypohidrotic ectodermal dysplasia and immunodeficiency
Source: Acta Neuropathol Commun. 2016 Jul 13;4:73. doi: 10.1186/s40478-016-0342-3 (PMC4944483; doi:10.1186/s40478-016-0342-3)
Supplement: Additional file 1: — Targeted sequencing strategy for BKV VP1 and NCCR regions. a. Products of nested PCR encompassing 1265 bp of BKV VP1 and 800 bp of NCCR with initial portion of early coding region are purified from an 1 % agarose gel and submitted to Sanger sequencing. b. Sanger sequencing results of VP1 and NCCR purified gel products. (PDF 427 kb) [file 40478_2016_342_MOESM1_ESM.pdf]

Supplementary Material

BK Virus Encephalopathy and Sclerosing Vasculopathy in a Patient with Hypohidrotic Ectodermal Dysplasia and Immunodeficiency

Acta Neuropathologica

Armine Darbinyan[1], Eugene O. Major[2], Susan Morgello[1, 3, 4], Steven Holland[5], Caroline Ryschkewitsch[2], Maria Chiara Monaco[2], Thomas P. Naidich[6], Joshua Bederson[7], Joanna Malaczynska[1], Fei Ye[1], Ronald Gordon[1], Charlotte Cunningham-Rundles[8], Mary Fowkes[1], Nadejda M. Tsankova[1,4]†

[1]Department of Pathology, Icahn School of Medicine at Mount Sinai, New York, NY 10029
[2]Laboratory of Molecular Medicine and Neuroscience, National Institute of Neurological Disorders and Stroke, National Institutes of Health, Bethesda, Maryland 20892
[3]Department of Neurology, Icahn School of Medicine at Mount Sinai, New York, NY 10029
[4]Department of Neuroscience and Friedman Brain Institute, Icahn School of Medicine at Mount Sinai, New York, NY 10029
[5]Laboratory of Clinical Infectious Diseases, National Institute of Allergy and Infectious Diseases, National Institutes of Health, Bethesda, MD 20892
[6]Department of Radiology, Icahn School of Medicine at Mount Sinai, New York, NY 10029
[7]Department of Neurosurgery, Icahn School of Medicine at Mount Sinai, New York, NY 10029
[8]Department of Medicine - Allergy & Immunology, Icahn School of Medicine at Mount Sinai, New York, NY 10029

† Corresponding Author

nadejda.tsankova@mssm.edu

Supplemental Figure S1 (Relates to Fig 4)

a

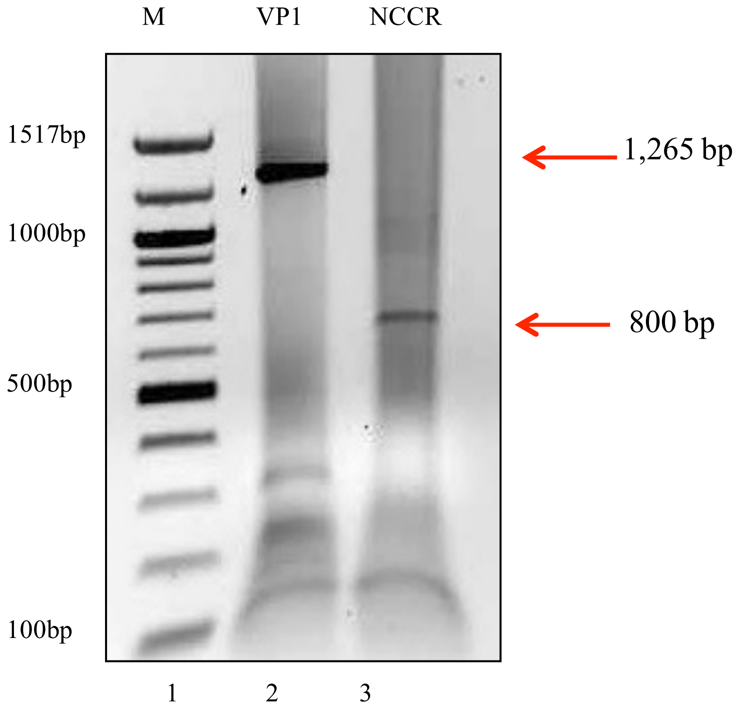

VP1 = 1,265 bp (Primers F: 1456-1475, R: 2725-2744)
NCCR = 800 bp (Primers F: 4881-4902, R: 567-588)
M - marker
Numerical positions of nucleotides in primers based on Dunlop strain, V01108

b

Sequencing results

<u>1. VP1 region of BKV</u> (2583-2154 nt)

5' –
AGGTCGTAGATAAAAGCAGCATTTATTAAGTCATGT
TTATTATACAATAAAAGCACCTTGTTTAAACCATTTT
TGTTTGCAATTGTCCCTGTCTGTCAATATATCTTATC
ATATCTGGGTCCCCTGGAAGCTGTTCTGTGCCATCAA
ACACCCTGACCTCCTCCATCTGATACTCCTGACCATA
CATAGGGGTCCCATCCACTCTCTGGGTTCTCCTGTTT
ATAAGGTCACTAAGCAAAAAGGAAATTGGGTAAGG
GTTCTTTACTTGATTTTTTCTCAGGCGAATCTTAAAA
TATCTTGGAAGGCCCCTCCACTGTTGTGTTCCAGAGC
TGTTAGTAAACAGTCCACAAATATCAGCAGCTGAAA
CATACAGGCTATCACTTTACACAGAGGCCCCCACCC
TGTTCATCCAGCAACACTGTGGTAGCTGTGTTGGTTA
C -3'

2. <u>Initial portion of early coding region and NCCR (red) of BKV</u>

5' –
TGGCACCTTTATACCTGCTCATTTTTTTATATAAAGT
ATTCATTCTCTTCTTTTATCCTCGTCGCCCCCTTTGTC
AGGGTGAAATTCCTTACACTTTCTTAAATAGGCTTT
CCTCATTAAGGGAAGGTTTCCCCAGGCAGCTCTTTC
AAGGCCTAAAAGGTCCATGAGCTCCATGGATTCCTC
CCTGTTTAGCACTTTATCCATTTTTGCAAAAAATTGC
AAAAGAATAGGGATTTCCCCAAATAGTTTTGCTAGG
CCTCAGAAAAAGCCTCCACACCCTTACTACTTGAGA
GAAAGGGTGGAGGCAGAGGCGGCCTCGGCCTCTTA
TATATTATAAAAAAAAAGGCCACAGGGAGGAGCTG
CTTTCCCATGGAATGCAGCCAAAGAGGAGCTGCTTT
CCCATGGAATGCAAATTCTCAAATAAACACAAAAT
AAAAAAAAAGGCCACAGGAAGGAGCTGCTTTCCCA
TGGAATGCACCCAAAGAGGAGCTGCTTTCCCATGG
AATGCAGCCCAACTATGCCCCCCGGCATGACTGGGC
AACCAAGCCCTGGCTGTTAATATTGAATCCCCGCCC
CGCAAATTAACAATAACCCCAATACGAAGTGGAAA
GTTGCCCTTGCCCCCACTTAGTATC – 3'

Fig S1 Targeted sequencing strategy for BKV VP1 and NCCR regions

a. Products of nested PCR encompassing 1265bp of BKV VP1 and 800 bp of NCCR with initial portion of early coding region are purified from an 1% agarose gel and submitted to Sanger sequencing. **b.** Sanger sequencing results of VP1 and NCCR purified gel products
